# Variations in diet composition of sympatric *Trachypithecus francoisi* and *Macaca assamensis* in the limestone habitats of Nonggang, China

**DOI:** 10.24272/j.issn.2095-8137.2018.046

**Published:** 2018-05-12

**Authors:** Qi-Hai Zhou, Zhong-Hao Huang, Hua Wei, Cheng-Ming Huang

**Affiliations:** 1 Key Laboratory of Ecology of Rare and Endangered Species and Environmental Protection, Ministry of Education; Guangxi Key Laboratory of Rare and Endangered Animal Ecology, Guangxi Normal University, Guilin Guangxi 541004, China; 2 Key Laboratory of Animal Ecology and Conservation Biology, Institute of Zoology, Chinese Academy of Sciences, Beijing 100101, China

**Keywords:** François’ langurs, Assamese macaques, Diet composition, Limestone habitat

## Abstract

Comparative studies of sympatric species are essential for understanding behavioral and ecological adaptation as well as the mechanisms that can reduce resource competition to allow coexistence. François’ langurs (*Trachypithecus francoisi*) and Assamese macaques (*Macaca assamensis*) are sympatric primate species found in the limestone seasonal rainforests of Nonggang Nature Reserve, southwestern Guangxi, China. To explore their different adaptation strategies, we collected data on diet using scan sampling at 15-min intervals. Our results revealed that François’ langurs showed a more flexible diet composition than Assamese macaques. François’ langurs increased dietary diversity and mature leaf consumption in response to seasonal scarcity of preferred young leaves and fruits, whereas Assamese macaques relied heavily on young bamboo leaves (*Indocalamus calcicolus*) in most months. These variations reflect the differences in digestive physiology, morphology, and the temporal and spatial distribution of food resources.

## INTRODUCTION

Comparative studies of sympatric species are essential for understanding behavioral and ecological adaptations, as well as the structure of animal communities (Fleagle et al., 1999; Fleagle, 2013). Many studies have revealed considerable variation in foraging strategies among sympatric primate species, with food type documented as one of the principal determining factors (Hadi et al., 2012; Nadjafzadeh & Heymann, 2008; Porter, 2001; Powzyk & Mowry, 2003; Singh et al., 2011; Tomblin & Cradford, 1994). For example, in the Peleonan forest of Sumatra, *Presbytis potenziani* feeds more selectively on fruits, whereas sympatric *Simias concolor* feeds predominantly on leaves (Hadi et al., 2012). In addition, sympatric primates can adopt different foraging strategies in response to temporal changes in food resources (Dammhahn & Kappeler, 2008; Porter, 2001; Standford, 2006; Thorén et al., 2011; Tutin & Fernandez, 1993). For example, gorillas (*Gorilla gorilla*) in Lopé Reserve, Gabon, rely on non-fruit foods when succulent fruits are scarce in the dry season, whereas sympatric chimpanzees (*Pan troglodytes*) continue to find and consume considerable quantities of fruit (Tutin & Fernandez, 1993). Many of these variations reflect differences in morphology, digestive physiology, and the temporal and spatial distribution of food resources (Hadi et al., 2012; Nadjafzadeh & Heymann, 2008; Powzyk & Mowry, 2003), and represent ecological niche separation, allowing the coexistence of sympatric primates.

The François’ langur (*Trachypithecus francoisi*) and Assamese macaque (*Macaca assamensis*) are sympatric species that reside on the limestone hills of southwestern Guangxi, China (Zhang et al., 2002). Despite differences in morphology and digestive system (Chivers, 1994), previous studies have documented similar food habits between the two species, with both found to be predominantly folivorous but with fruit consumed when available (Ahsan, 1994; Chalise, 2003; Hu, 2011; Zhou et al., 2006). Thus, these similarities imply the possibility of food competition between the two species. To date, however, no comparative studies have reported on the foraging strategies of sympatric François’ langurs and Assamese macaques, which is important for understanding their coexistence mechanisms.

In this paper, we compared the diet of François’ langurs and Assamese macaques living sympatrically in the limestone habitats of southwestern Guangxi, China. We aimed to: (1) determine how food resources differ between them; (2) investigate how they adjust behavior in response to seasonal changes in food resources; and (3) explore whether differences in foraging strategies can explain their coexistence. 

## MATERIALS AND METHODS

### Study sites and subjects

**Figure 1:**
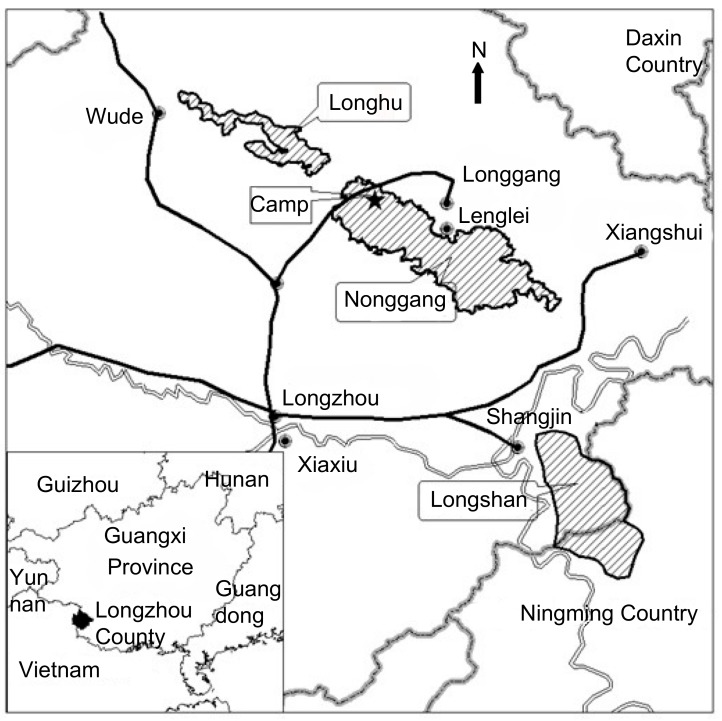
Map of Nonggang Nature Reserve showing the study site and surrounding area

This study was conducted from October 2005 to September 2006 in Nonggang Nature Reserve (E106°42′–E107°4′, N22°13′–N22°33′, Figure 1), Guangxi Province, China. The reserve comprises three areas, Nonggang (5 426 hm^2^), Longhu (1 034 hm^2^), and Longshan (3 949 hm^2^), which are separated by farmland and villages. The reserve consists of limestone hills ranging from 400 m to 600 m a.s.l. (Guangxi Forest Department, 1993). The vegetation is characterized by limestone seasonal rainforest. Annual precipitation was 1 373 mm (October 2005–September 2006) and 952 mm (October 2006–September 2007). There are two distinct seasons: a rainy season from April to September with >80 mm monthly rainfall and a dry season in the remainder of the year with <80 mm monthly rainfall (Zhou et al., 2007). 

Our study site is found within the northwestern portion of Nonggang (Figure 1). We selected one group of François’ langurs (*n*=9) and two groups of Assamese macaques (Group 1, *n*=15; Group 2, *n*=12) who ranged nearest to our temporary camp. The home ranges of the three study groups overlapped with each other. The François’ langur group consisted of one adult male, five adult females, and three infants. Assamese macaque Group 1 consisted of two adult males, four adult females, four adult individuals of unidentified sex, and five juveniles, and Group 2 consisted of two adult males, four adult females, two adult individuals of unidentified sex, and four juveniles.

### Ecological sampling

We conducted vegetation surveys in the main study area at the onset of behavioral data collection. We used a stratified random sampling method for the placement of vegetation plots. We placed 13 plots (50 m×10 m) in the main study area, including four in valley basins and nine on hillsides. The plots covered most vegetation types described by Shu et al. (1988). Within the plots, all trees with ≥5 cm diameter at breast height were tagged. In total, we monitored 312 trees from 30 families at monthly intervals, and recorded the presence of young leaves, fruits, and flowers. The relative abundance of different plant parts was expressed as a percentage of trees bearing the plant parts of interest each month, regardless of the size of the canopy. Huang et al. (2010) described monthly phenology changes.

### Data collection

We conducted behavioral observations of the François’ langur group for 126 d (7–22 d each month) and of the Assamese macaque groups for 58 d (3–9 d each month). Each day, data collection began when the monkeys were first encountered, and ended when they disappeared or entered their sleeping sites. We used scan sampling with 15-min intervals. Each scan lasted 5 min, followed by 10 min of inactivity until the next scan began. We recorded the activity of each individual seen during each scan. We watched each individual for 5 s after detection. The behaviors included four activity categories: resting, moving, feeding, and social behavior (e.g., grooming and playing). To avoid sampling bias toward certain individuals or a particular age-sex class, we collected behavioral records on as many different individuals as possible during a scan so that all individuals in the focal group were included, but we sampled no individual more than once. When the individual was feeding, we recorded plant species and parts eaten, e.g., young leaf, mature leaf, fruit, flower, seed, or other (e.g., petiole and stem). During the study period, a total of 8 168 behavior records were obtained from 3 052 scan samples for François’ langurs, which included 1 599 feeding records. We collected 6 525 behavior records from 1 666 scan samples for Assamese macaques, which included 1 259 feeding records. 

### Data analysis

Because few records were collected in September or October 2005 for the Assamese macaque groups, we excluded data in these months from later analyses. We also excluded records for dependent infants and juveniles because these animals were not acting independently. We determined the percentage of different plant species in the diet of each study group by calculating the percentage of feeding records devoted to them among annual total feeding records. We calculated the Shannon-Weaver diversity and evenness indices based on consumption of plant species. The Shannon-Weaver diversity index was calculated using the equation: *H*’=−∑*P_i_*×ln*P_i_* (where *P_i_* is the proportion of feeding records of the *i*th plant species) and the evenness index was calculated using the equation: *J*=*H*’/*H*_max_=*H*’/ln*n* (where *n* is the number of species eaten). Food category composition was expressed as the percentage of different plant parts in the monthly diet of the study group using monthly total feeding records. Annual food category composition was obtained by averaging the monthly percentages.

We used the Wilcoxon signed-rank test to examine inter-specific variations in the overall pattern of use of different food categories. The Mann-Whitney *U* test was used to examine seasonal variations in the use of different food categories. We used Spearman rank correlations to test the relationship between the abundance and consumption of different plant parts. All tests were two-tailed, with a significance level of 0.05.

### RESULTS

During the study period, we identified 92 plant species consumed by François’ langurs and 69 plant species consumed by Assamese macaques. Major foods (accounting for >1% of total feeding records) contributed to a large proportion of the total diet (François’ langur: 80.6%; Assamese macaque: 85.2%, Table 1). However, the François’ langurs used more plant species as major foods than did the Assamese macaques. Annual diversity and evenness of use of major food plants were higher for François’ langurs (*H*=2.563, *J*=0.817) than for Assamese macaques (*H*=1.164, *J*=0.468). This indicated that Assamese macaques concentrated on fewer food species than François’ langurs: the top 10 food species accounted for 82.8% of the Assamese macaque diet, but only for 54.3% of the François’ langur diet. In particular, *Indocalamus calcicolus*, a small bamboo species, contributed to 62% of the annual diet of Assamese macaques.

**Figure 2:**
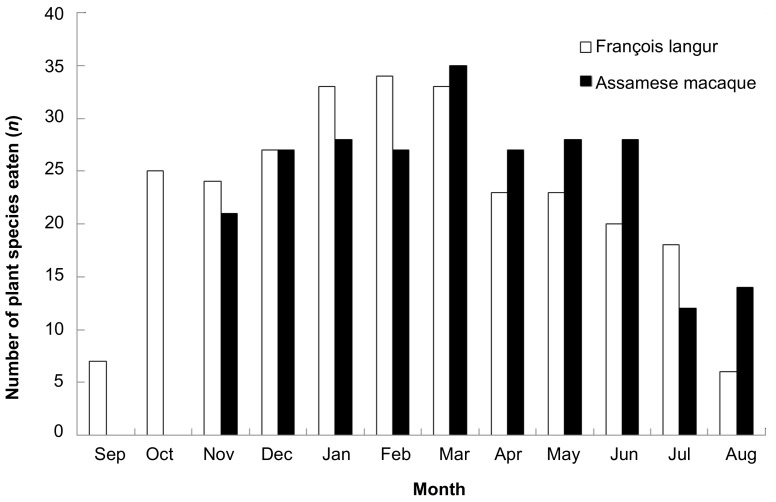
Monthly number of plant species eaten by sympatric François’ langurs and Assamese macaques at Nonggang

There was no significant difference in monthly number of plant species eaten by François’ langurs and Assamese macaques (*Z*=−0.356, *n*=10, *P*=0.722, Figure 2). However, inter-specific differences in seasonal dietary diversity in response to food availability were observed. François’ langurs consumed more plant species in the dry season than in the rainy season (*U*=4.000, *n_1_*=6, *n_2_*=6, *P*=0.025). Monthly number of plant species eaten by langurs correlated negatively with monthly fruit availability (*r_s_*=−0.840, *n*=12, *P*=0.001). For Assamese macaques, there was no significant seasonal difference in monthly number of food species (*U*=9.000, *n_1_*=5, *n_2_*=5, *P*=0.548).

**Figure 3:**
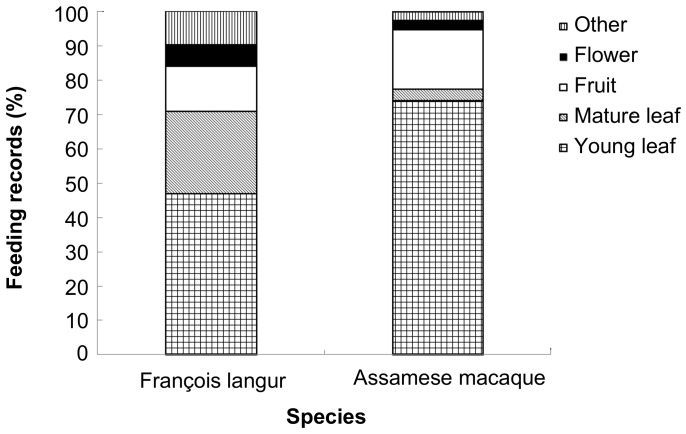
Annual diet composition of sympatric François’ langurs and Assamese macaques at Nonggang

**Figure 4:**
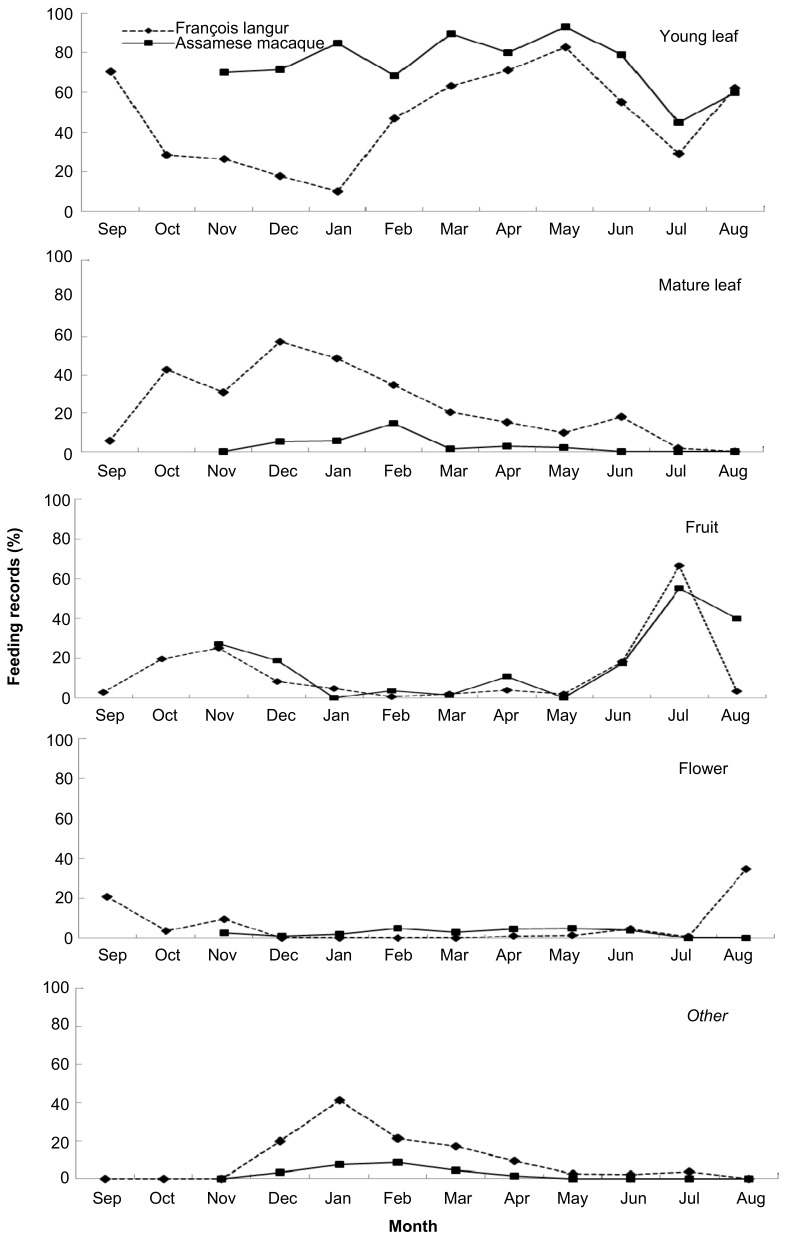
Monthly percentage of feeding records devoted to different food items in the diet of sympatric François’ langurs and Assamese macaques at Nonggang

**Figure 5:**
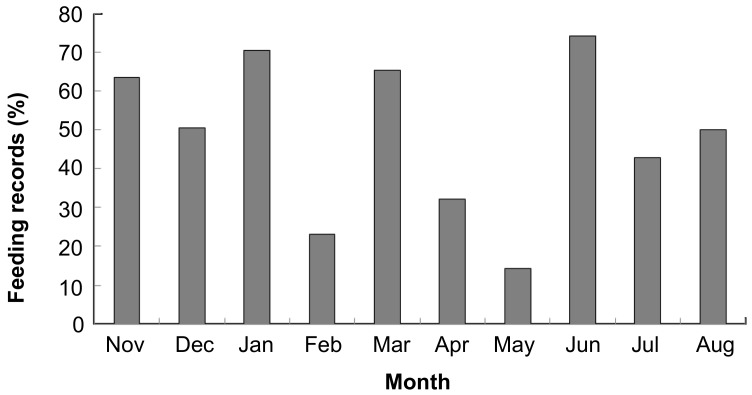
Monthly percentage of feeding records devoted to young leaves of *Indocalamus calcicolus* by Assamese macaques at Nonggang

In general, both species were highly folivorous, with leaves accounting for 71% and 77.4% of the overall diet for François’ langurs and Assamese macaques, respectively (Figure 3). Fruits contributed to only a small proportion of the overall diet (17.4% for François’ langurs and 13.4% for Assamese macaques). However, there was marked inter-specific variation in annual food category composition (Figure 3). Assamese macaques ate more young leaves (*Z*=−2.701, *n*=10, *P*=0.007), whereas François’ langurs consumed more mature leaves (*Z*=−2.666, *P*=0.008) and other items (e.g., seeds and petioles, *Z*=−2.521, *P*=0.012). Moreover, inter-specific variation in seasonal dietary shift in response to food availability was detected. François’ langurs consumed more young leaves in the rainy season than in the dry season (*U*=4.000, *n_1_*=6, *n_2_*=6, *P*=0.025, Figure 4). The consumption of young leaves correlated positively with their abundance (*r_s_*=0.865, *n*=12, *P*<0.001). Conversely, the consumption of mature leaves (*U*=0.000, *P*=0.004) was higher in the dry season than in the rainy season. A significant and negative correlation was found between the consumption of young leaves and mature leaves (*r_s_*=−0.685, *n*=12, *P*=0.014). In addition, langurs consumed seeds only in the dry season (*U*=6.000, *P*=0.022), and the proportion to monthly diet varied from 6% (March) to 22% (January). For Assamese macaques, young leaves contributed to the highest proportion of the monthly diet almost year-round (Figure 4). In contrast to François’ langurs, Assamese macaques consumed more young leaves in the dry season than in the rainy season, even though the difference was not statistically significant (*U*=11.000, *n_1_*=5, *n_2_*=5, *P*=0.754). It is worth noting that young leaves of *Indocalamus calcicolus* contributed to the bulk of the macaques’ total diet (Table 1) and young leaf consumption in most months (Figure 5). Macaques increased the consumption of this item in the dry season compared with the rainy season, but the difference was not statistically significant (*U*=8.000, *P*=0.421).

**Table 1:** Plant species used as major foods by sympatric François’ langurs and Assamese macaques at Nonggang.

François’ langur					Assamese macaque			
Species	Family	Part^a^	F% ^b^		Species	Family	Part^a^	F% ^b^
*Ficus nervosa*	Moraceae	YL,ML,F,FR	9.39		*Indocalamus calcicolus*	Bambusoideae	YL	62.02
*Pithecellobium clypearia*	Mimosaceae	YL,ML,S	7.82		*Ficus nervosa*	Moraceae	YL,ML,F,FR	4.01
*Aristolochia longgangensis*	Aristolochiaceae	YL,ML,ST	5.94		*Guihaia argyrata*	Palmae	F,P	3.64
*Canthium dicoccum*	Rubiaceae	YL,ML,F,FR	5.76		*Sinosideroxylon pedunculatum*	Sapotaceae	YL,FR	3.55
*Embelia scandens*	Myrsinaceae	YL,ML	5.57		*Canthium dicoccum*	Rubiaceae	YL,ML,F,FR	2.28
*Ventilago calyculata*	Rhamnaceae	YL,ML,F,FR	4.57		*Burretiodendron hsienmu*	Tiliaceae	YL	1.55
*Ficus glaberrima*	Moraceae	YL,ML,FR	4.26		*Croton euryphyllus*	Euphorbiaceae	YL	1.55
*Pueraria thunbergiana*	Papilionaceae	YL,ML	4.13		*Ficus microcarpa*	Moraceae	YL,FR	1.46
*Wrightia pubescens*	Apocynaceae	YL,ML,F,S	3.50		*Ventilago calyculata*	Rhamnaceae	YL	1.37
*Clausena anisum*	Rutaceae	FR	3.32		*Berchemia floribunda*	Rhamnaceae	YL,F,P	1.37
*Zenia insignis*	Caesalpiniaceae	YL	3.19		*Lepionurus sylvestris*	Sapotaceae	YL,ML	1.18
*Pseudostreslus indica*	Moraceae	YL,ML	2.88		*Sapium rotundifolium*	Euphorbiaceae	FR	1.18
*Sabia swinhoei*	Sabiaceae	YL,ML	2.57					
*Randia spinosa*	Rubiaceae	YL,ML	2.50					
*Sinosideroxylon pedunculatum*	Sapotaceae	YL,FR	2.32					
*Urobotrya latisquama*	Opiliaceae	YL,ML,F	2.07					
*Cuscuta chinensis*	Convolvulaceae	YL,ML	2.00					
*Tirpitzia ovoidea*	Linaceae	YL,ML,F	1.75					
*Burretiodendron hsienmu*	Tiliaceae	YL	1.69					
*Ficus gibbosa*	Moraceae	YL,ML,FR	1.63					
*Boniodendron minor*	Sapindaceae	YL,ML	1.50					
*Teonongia tonkinensis*	Moraceae	YL,ML	1.25					
*Cudrania cochinchinensis*	Moraceae	YL,ML	1.01					

^a^Parts eaten: YL, young leaf; ML, mature leaf; FR, fruit; S, seed; F, flower; P, petiole; ST, stem. ^b^F%: Percentage of total feeding records.

## DISCUSSION

In this study, both primates exhibited marked differences in their choice of food species. Compared to François’ langurs, Assamese macaques concentrated more foraging effort on a few food species, especially *Indocalamus calcicolus*, which accounted for 62% of their diet. This small bamboo is superabundant and endemic to limestone hills (Liang et al., 1988). Thus, using more readily available common plant species as a food source may be an effective strategy for Assamese macaques to survive in limestone habitats. In contrast, François’ langurs showed more flexibility in food choice, as reflected in their higher dietary diversity and evenness indices. Moreover, François’ langurs tended to select less common plant species as favored foods. Of the top 10 food species, only two (*Pithecellobium clypearia*, *Clausena anisum*) belonged to the dominant tree species in the habitat (Zhou et al., 2006). 

Although both primates were highly folivorous, they exhibited dietary differences: François’ langurs consumed more mature leaves and less young leaves than Assamese macaques. These variations may reflect differences in feeding strategies in response to changing food availability. In typical colobine species diets, young leaves and fruits are often preferred foods (Oates, 1994; Yeager & Kool, 2000). When young leaves and fruits became scarcer in the dry season, François’ langurs significantly increased their consumption of mature leaves. Mature leaves are abundant and available, but rich in cellulose and poor in nutrition (Richard, 1985). They usually serve as fallback foods for primates during periods of preferred food scarcity (Marshall & Wrangham, 2007). In contrast, Assamese macaques maintained a high level of young leaves in the diet almost year-round, even an increase in young leaf consumption in the dry season. This could be related to the fact that Assamese macaques relied heavily on young leaves of *Indocalamus calcicolus*, which contributed to the bulk of their diet in most months and exhibited a similar tendency to young leaf consumption. Young leaves of this bamboo species are plentiful throughout the year, and their availability is invariant between seasons (personal observation), which may provide a long-term staple food resource for the macaques in limestone habitats. Thus, the bamboo-dominated diet could be a key factor in the differences in fallback foods between Assamese macaques and François’ langurs.

As colobines, langurs possess digestive systems distinct from the cercopithecine macaques. Langurs have an enlarged and sacculated forestomach, which serves as the primary fermentation chamber (forestomach fermentation) (Chivers, 1994). In contrast, macaques have an enlarged caecum or colon as the primary fermentation chamber (caecocolic fermentation) (Chivers, 1994; Hladik, 1978). The former is most efficient for mammals that rely on foods high in structural carbohydrates, whereas the latter is most efficient for mammals that primarily feed on readily digestible foods (Alexander, 1993; Lambert, 1998). Thus, François’ langurs can consume mature leaves in large quantities as fallback foods in response to preferred food scarcity, whereas Assamese macaques depend more on young leaves. 

In addition to digestive physiology, dietary variation may be related to differences in positional behavior reflected in anatomy (Chalise, 2003; Fleagle, 2013; McGraw, 1998). It is likely that different postural abilities enable species to exploit different food resources. Although sitting is the most common feeding posture for both primate species, Assamese macaques adopted the stank/forelimb-suspended posture more frequently than François’ langurs during feeding (unpublished data for François’ langurs, Huang et al., 2015). The stank/forelimb-suspended posture was used most frequently when Assamese macaques fed on young leaves of *Indocalamus calcicolus*. Macaques always stand bipedally on the ground and grasp the stem with one forelimb while they pluck young leaves with the other .

In summary, sympatric François’ langurs and Assamese macaques at Nonggang adopted different foraging strategies in response to changes in the temporal and spatial distribution of preferred foods, which appears to reduce competition for food resources and allows for sympatry. 
